# Central venous pressure line insertion for the primary health care physician

**DOI:** 10.4102/safp.v65i1.5740

**Published:** 2023-06-08

**Authors:** Indiran Govender, Henry I. Okonta, Olukayode Adeleke, Selvandran Rangiah

**Affiliations:** 1 Department of Family Medicine and Primary Health Care, Faculty of Health Sciences, Sefako Makgatho Health Sciences University, Pretoria, South Africa; 2 Department of Family Medicine and Primary Health Care, School of Medicine, Sefako Makgatho Health Sciences University, Pretoria, South Africa; 3 Department of Family Medicine and Rural Health, Faculty of Health Sciences, Walter Sisulu University, Mthatha, South Africa; 4 Department of Family Medicine, Faculty of Health Sciences, University of KwaZulu-Natal, Durban, South Africa

**Keywords:** internal jugular vein, primary health care physician, subclavian vein, resuscitation, intra vascular assess, anatomy

## Abstract

Central venous access is an important procedure to understand and perform not only in the emergency unit but also for prolonged reliable venous access. All clinicians must be familiar and confident with this procedure. This paper will focus on applied anatomy in respect of common anatomical sites for venous access, the indications, the contraindications, the technique and complications that may arise following the procedure. This article is part of a series on vascular access. We have previously written on the intra osseous procedure and an article on umbilical vein catheterisation will follow.

## Introduction

Central venous catheterisation is a common procedure whereby a central venous catheter is placed into a large central vein to facilitate various venous interventions and device insertions.^1,2^ It is widely used in emergency settings or for prolonged intravenous therapies for reliable venous access.^3,4^ This method of catheter placement into central vein lumens was first introduced by Werner Forssman in 1929, a procedure for which he was awarded the Nobel Prize in 1956.^5,6,7^ The technique was adapted by Sven Ivar Seldinger in 1953 and has remained a widely used and valuable procedure that has contributed significantly to the development of modern medicine for both stable and critical patients. The Seldinger technique, as it is commonly referred to, remains an essential skill and an indispensable clinical tool for healthcare practitioners.^8,9^ With new catheter designs, standardised techniques and ultrasound guidance, this catheter placement procedure over the decades has witnessed remarkable user improvement and reduced complications.^8^

## Applied anatomy

**FIGURE 1:**
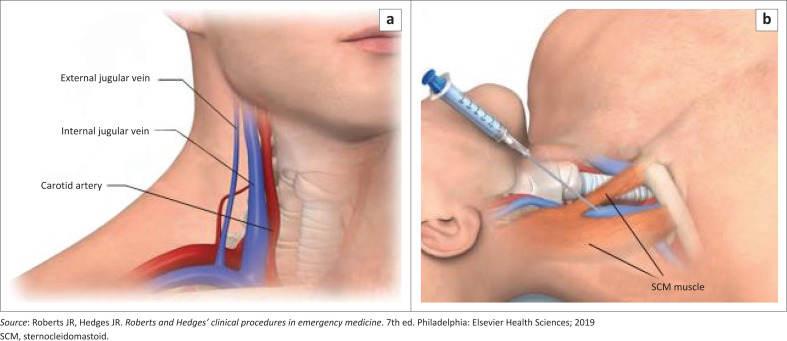
Internal jugular vein anatomy and needle placement.

**FIGURE 2:**
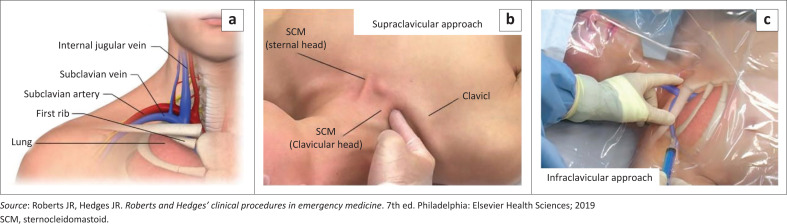
Subclavian vein anatomy and needle placement.

**FIGURE 3:**
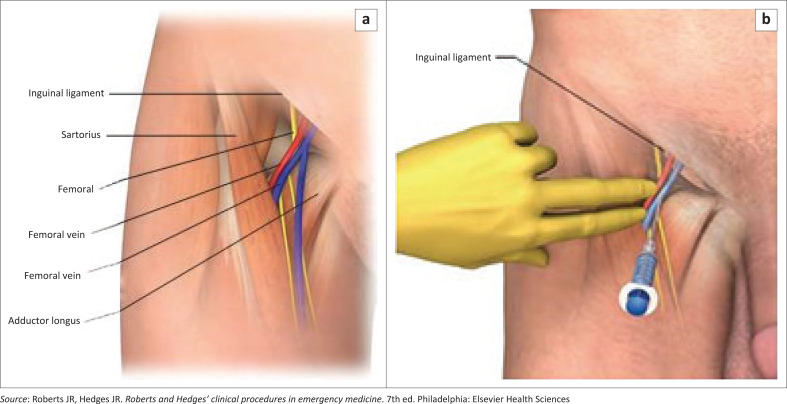
Femoral vein anatomy and needle placement.

The three main access sites for central venous catheterisation are the internal jugular vein, subclavian vein and the common femoral vein (Figure 1, Figure 2 and Figure 3).^10^ Other sites are the basilic and brachial veins that are used for peripherally inserted central vein catheters. Each anatomical site has its relative risks and benefits, and the choice of site for central venous catheterisation is based on indication for placement, patient vascular anatomy, clinician experience and preference.^1^

The internal jugular vein has the advantage of a reliable anatomy, accessibility and low complication rates.^11^ It has the added advantage of being suitable for ultrasound guidance use.^11^ In the absence of any compelling clinical parameters, the right internal jugular vein is preferable to the left internal jugular vein because it forms a more direct path to the superior vena cava with a wider calibre and has a more superficial location that makes it easier to approach.^12^ Some clinical scenarios that may compel the use of the left internal jugular vein include trauma, neck cancer or invasive devices on the right side. The internal jugular vein is located between the two heads of the sternocleidomastoid muscle at the base of the neck where it lies anterolateral to the common carotid artery (Figure 1). It courses forward to confluence with the subclavian vein to form the brachiocephalic vein that from the left and right join to form the superior vena cava.^13,14^

The subclavian vein offers the advantage of being accessible when the application of a cervical collar in trauma scenarios preclude access to the internal jugular vein. It also has low rates of infectious and thrombotic complications.^15^ The disadvantages for the subclavian vein site are the higher rates of pneumothorax, the location behind the clavicle that renders it noncompressible and less accessible for ultrasound-guided catheter placement. The subclavian vein site is located 2 cm – 3 cm inferior to the midpoint of the clavicle that is 1 cm – 2 cm lateral to the curvature of the clavicle^8^ (Figure 2).

The common femoral vein is easily compressible, which gives it an advantage in trauma and coagulopathic patients. In some critically ill patients with applied resuscitation equipment and devices for airway access, this site remains free and available for central venous access.^1^ Iatrogenic pneumothorax is not a concern at this site. The common femoral site has the disadvantage of increased thrombotic complications and does not allow for accurate central venous pressure measurement.^1,16^ The common femoral vein is located in the femoral triangle, which is demarcated by the inguinal ligament superiorly, sartorius muscle laterally and the adductor longus medially. It lies medial to the femoral artery, which serves as an anatomic landmark to find the vein (Figure 3).

Some of the advantages and disadvantages of the three main access sites are shown in Table 1.

**TABLE 1:** Advantages and disadvantages of central venous access sites.

Approach	Advantages	Disadvantages
Internal jugular vein	Good external landmarks Improved success with ultrasound Less risk for pneumothorax than with subclavian access Can recognise and control bleeding Malposition of the catheter is rare Almost a straight course to the superior vena cava on the right side Carotid artery easily identified	More difficult and inconvenient to secure Possibly higher infectious risk than with subclavian access Possibly higher risk for thrombosis than with subclavian access Risk of carotid artery puncture
Subclavian vein	Good external landmarks Practical method of inserting a central line in cardiorespiratory arrest Accessible when the application of cervical collar precludes access to the internal jugular vein Has low rates of infection and thrombotic complications^15^	Increased risks of pneumothorax Unable to compress bleeding vessels Less accessible for ultrasound guided catheter placement
Common femoral vein	Good external landmarks Easily compressible during ultrasound technique requiring less cooperation from patient and less positioning Useful alternative with trauma and coagulopathy^1^ No interference with CPR No risk of pneumothorax	Increased thrombotic complications^16^ Not reliable for CVP measurement^1^ Difficult to secure in ambulatory patients

*Source:* Roberts JR, Hedges JR. *Roberts and Hedges’ clinical procedures in emergency medicine*. 7th ed. Philadelphia: Elsevier Health Sciences

CPR, cardiopulmonary resuscitation; CVP, central venous pressure.

## Indications

The indications for central venous catheterisation include the following:

- Drug infusions that may be incompatible with peripheral intravenous access such as vasopressors, hyperosmolar solutions, total parenteral nutrition, high concentration of potassium chloride and chemotherapy agents.
- Inability to obtain adequate peripheral venous access in emergency situations.
- Monitoring central venous pressure, pulmonary artery pressure and central venous oxyhemoglobin saturation.
- Transvenous pacing wire placement.
- High-volume flow procedures with large-bore access as in hemodialysis, plasmapheresis and extracorporeal membrane oxygenation.
- Vena cava filter placement.
- Pulmonary artery catheter placements.

## Contraindications

The literature is not clear as to the existence of absolute contraindications for central venous catheter placement, and relative contraindications are dependent on the urgency and accessibility of alternative venous sites.^1,4,8^

Site-specific contraindications include the following:

- skin or soft tissue infection at the insertion site
- anatomical distortion at the site
- vascular injury proximal or distal to the site of insertion
- thrombus within the intended vein.

Relative contraindications include the following:

- coagulopathy
- thrombocytopenia
- morbid obesity.

Patients with international normalised ratio greater than 3.0 or platelets less than 20 × 10/L have increased risk of bleeding^17^ and should be considered for pre-procedure administration of appropriate blood products (fresh frozen plasma, platelets).^1,4^

## Equipment

There are several types of central venous catheters and devices, the use of which depends on clinical parameters (acute, subacute, chronic), duration of use (short term, medium term, long term), type of insertion (central or peripheral), location (peripheral or central) and number of lumens (single, double, triple).^2^

**FIGURE 4:**
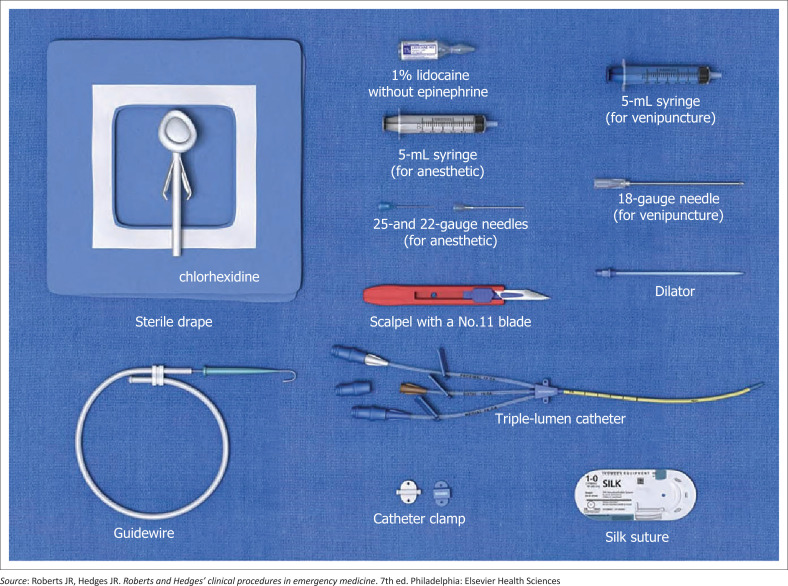
Equipment used to insert the CVP.

Equipment for central venous catheter placement include the following (see Figure 4):

- sterile cleansing solution (chlorhexidine)
- sterile gauze
- syringes and needle for local anaesthetic and sterile saline flush
- local anaesthetic – 1% Lidocaine
- introducer needle with syringe
- sterile saline
- scalpel
- guide wire
- tissue dilator
- appropriate suture material with needle
- sterile gloves
- sterile dressing
- central venous access kit (triple-lumen, dual-lumen or large-bore single lumen venous catheter).

### Intravenous tubing and connectors

Ultrasound machine with sterile ultrasound transducer gel and sterile ultrasound probe if available.

### Preparation for the procedure

Informed consent for the procedure is obtained with the risks, benefits and potential complications discussed and explained to the patient. In emergency situations where this is not possible, consent is implied.

Pre-cannulation vein assessment by bedside ultrasonography (where available) is recommended to ascertain relevant anatomy and to select the most appropriate site for the patient.^18^

Performing the procedure under ultrasound guidance is currently the standard of care, as it has improved success rates, decreased the number of punctures per attempt as well as complications, compared to the use of landmarks only.^19^ Although ultrasound makes it safer in general, it is dependent on the skill and training of the clinician concerned and knowing how to figure out your landmarks on ultrasound.

When using the ultrasound probe, it is conventional that the clinician stands on the right side of the patient and the probe marker faces towards the patient’s head or the patient’s right side. This ensures the probe marker on the left side of the screen is properly aligned with the clinician’s left side and when moving the probe to the left or right the images on the screen will move in that direction as well.^19^

In patients with unilateral lung disease, the ipsilateral side to the compromised lung is recommended for venous access.^2^

The patient is put in the appropriate position for the selected site. For internal jugular and subclavian access sites, patient is put in a 15-degree Trendelenburg position if clinical condition permits. This position engorges the vessel and improves the chance of first-pass success. It also minimises the risk of venous air embolism during the procedure.^20^ If a C-spine injury is not suspected, turn the patient’s head away from the side of cannulation to better outline the anatomical landmarks.^21^ It is recommended that the patient’s arm is placed in the adduction position against the torso while the shoulder is kept in a neutral to lower shoulder position. This allows for a consistent landmark by increasing the area of contact between the subclavian vein and the undersurface of the clavicle.^14^ In the common femoral vein access approach, the patient should lie supine. For all sites, the patient should be placed on continuous cardiac monitor and pulse oximetry.

A sterile technique is followed with full barrier precautions that includes surgical antiseptic handwash, long-sleeved sterile gown, surgical mask, gloves and cap.^21,22^ The skin at the selected site is cleaned with antiseptic solution, preferably more than 0.5% chlorhexidine.^23^ The patient is covered with sterile drapes large enough to cover the entire body but exposing the cleaned access site.

### Procedure^1,8^

**FIGURE 5:**
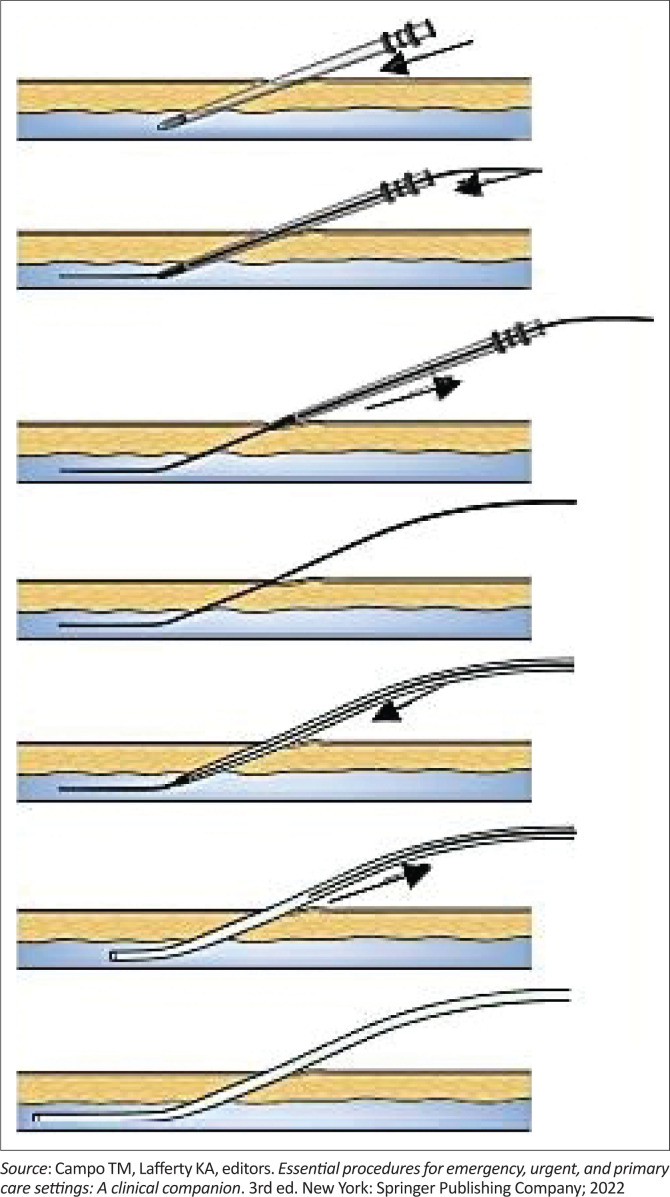
Seldinger guidewire technique.

It involves using a finder needle to identify the vessel, inserting a guide wire into the vessel through the finder needle, placing a catheter over the proximal end of the guide wire and inserting the catheter distally into the vessel in a progressive manner, finally removing the guide wire after the catheter has been advanced deep into the vessel^19^ (Figure 5).

**FIGURE 6:**
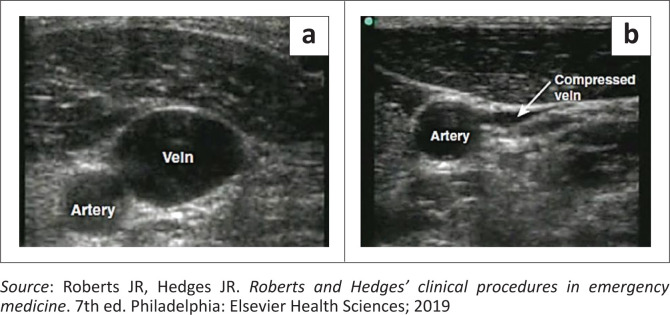
Ultrasound appearance of artery and vein. (a) Cross-sectional view of the artery and uncompressed vein. (b) Cross-sectional view of the artery and compressed vein.

Identify the vein under ultrasound guidance. The vein will appear round with a black centre that is compressible under pressure from the probe as shown in Figure 6.^10^ If ultrasound machine is not available, then landmark techniques as described earlier are used.

**FIGURE 7:**
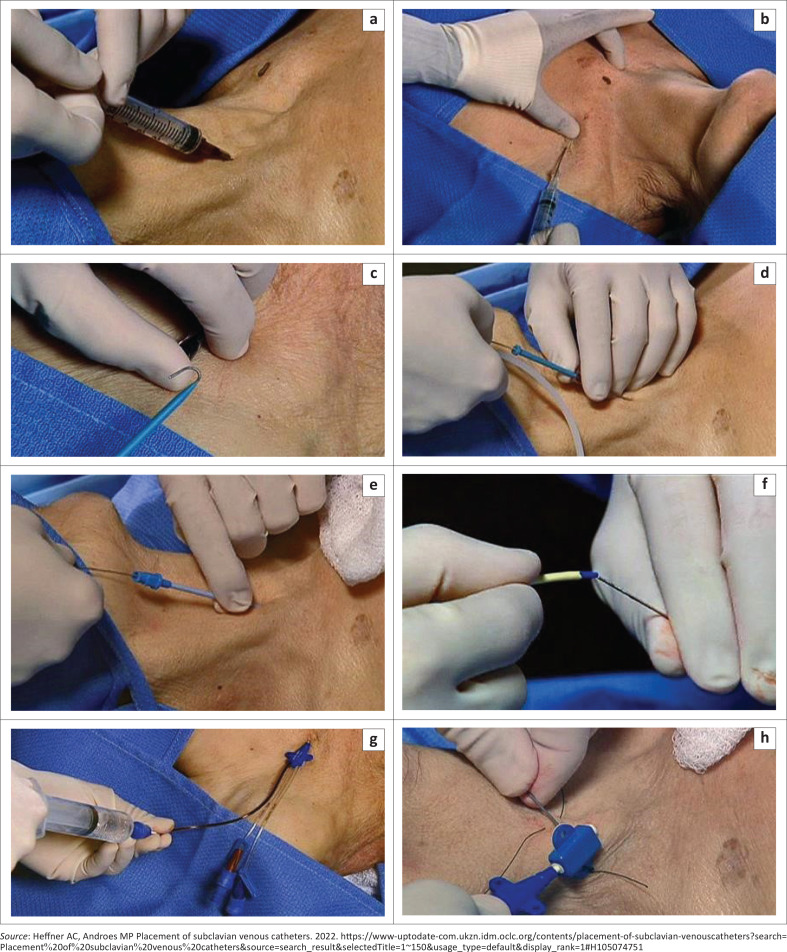
Steps in central venous catheterisation. (a) Finder needle supraclavicular approach, (b) infraclavicular approach, (c) guidewire with J tip, (d) placement of guidewire, (e) insertion of tissue dilator, (f) catheter threaded over guidewire, (g) aspiration following catheter placement and (h) catheter secured by suturing.^14^

Inject the local anaesthetic agent at the site of insertion. Insert the introducer needle attached to a 10-mL syringe with negative pressure while slowly advancing the needle into the vein (Figure 7a and b). Stop advancing the needle once blood is aspirated. Remove the syringe and occlude the needle to prevent air embolism.

Some CVP kits have syringes with an insertion point for the guidewire into the syringe and needle thereby limiting additional movement and hence minimising the risk of losing control of the needle.

Thread the guide wire through the introducer needle hub while observing the cardiac monitor for arrhythmia (Figure 7d). Remove the introducer needle hub while holding the guide wire to avoid disappearance of the guide wire into the vein.

Make a nick with the scalpel in the skin adjacent to the guide wire, and feed the dilator over the guide wire into the subcutaneous tissue with a twisting movement (Figure 7e). The dilator should be grasped and pushed close to the skin to prevent kinking. Remove the dilator and thread the central venous catheter over the guide wire until the guidewire emerges from the distal port. The guidewire should be grasped before catheter advancement (Figure 7f).

Remove the guide wire once the central venous catheter is in place. Cover the open port with the thumb until the end cap is screwed on. Flush and aspirate each lumen with sterile saline (Figure 7g).

Auscultate the chest to confirm equal bilateral breath sounds. Suture the catheter to the skin and apply sterile dressing (Figure 7h).

Before infusing intravenous (IV) fluids, lower the IV fluid reservoir to below the level of the patient’s right atrium and check the line for backflow of blood, which is suggestive but not diagnostic of intravascular placement. A pulsatile blood column may be noted if the catheter has been inadvertently placed in an artery. Intravascular placement can also be checked by attaching a syringe directly to the catheter hub and aspirating venous blood. It is also advisable to ensure that the catheter is easily flushed with a saline solution. X-rays are always indicated to verify catheter location and assess for potential complications such as pneumothorax or haemothorax.

Pull the catheter back slightly if the tip is too deep, in the right atrium or right ventricle.

The venous blood gas and central venous pressure obtained from the distal port of the catheter are optional means to confirm correct placement of the central venous catheter in addition to fluoroscopy and transoesophageal echocardiography.

Other confirmation techniques include saline flush test, post procedure efast for pneumothorax and visualisation of the wire in the vein and catheter in the vein using ultrasound.

### Post-insertion management

The central venous catheter device should be checked daily for patency. The access site should be inspected daily for bleeding, hematoma and signs of infection such as warmth, erythema and purulent discharge. Soiled dressings must be changed, and a sterile procedure must be used for any manipulation of the catheter site. The injection ports, catheter hubs and needleless connectors must be disinfected with antiseptics, and the intravenous administration sets should be changed regularly.^1^

There should be daily review of the need for the central venous catheter, and, if no longer necessary, the catheter must be removed. The common femoral central venous catheters should not be kept for more than 24 h – 48 h as they are prone to infection and make ambulation difficult.^8^

### Complications

The complications that can occur from central venous catheter placement can be procedural (complications because of the catheter placement procedure) or post-procedural (complications because of the indwelling catheter equipment).

The procedural complications include the following:

- arrhythmias, usually ventricular arrhythmias or bundle branch blocks, because of irritation of the atria or ventricles from placement of the guide wire and catheter tip beyond the atriocaval junction
- arterial puncture and bleeding
- catheter misplacement because of anatomical variation or distortion of the catheter site^23^
- pneumothorax
- venous air embolism.

The post-procedural complications are as follows:

- catheter-related bloodstream infections
- catheter-related thrombosis
- central vein stenosis
- venous catheter occlusion by kinks, backwash of blood or infusion of insoluble substances that form precipitates.

## Conclusion

Intravenous access through central catheters ensures hemodynamic monitoring, blood sampling, reliable access points for temporary and permanent cannulations and device placement. The internal jugular, subclavian and the common femoral veins are the three main access sites for central venous catheterisation, and practitioners should be familiar with the basic anatomy of these sites. The procedure should ideally be performed under ultrasound guidance to reduce complications.
